# Comparing the efficacy of mydriatic cocktail-soaked sponge and conventional pupil dilation in patients using tamsulosin – a randomized controlled trial

**DOI:** 10.1186/1471-2415-13-83

**Published:** 2013-12-20

**Authors:** János Hargitai, László Vezendi, Jørgen Vigstrup, Finn Eisgart, Søren Lundbye-Christensen, Bálint Hargitai, Henrik Vorum

**Affiliations:** 1 Department of Ophthalmology, Thy-Mors Hospital, Højtoftevej 2, Thisted 7700, Denmark; 2 Department of Cardiology, Aalborg University, Aalborg, Denmark; 3 UK Specialist Hospitals, Emersons Green NHS Treatment Center, Bristol, UK; 4 Department of Ophthalmology, Aalborg University, Aalborg, Denmark

**Keywords:** Pupil dilation, Intraoperative floppy iris syndrome, Tamsulosin

## Abstract

**Background:**

A strong association exists between the use of tamsulosin and the occurance of intraoperative floppy iris syndrome. Several methods were advocated to overcome the progressive intraopertive miosis.

Our purpose was to investigate the effect of a mydriatic-cocktail soaked cellulose sponge on perioperative pupil diameter in tamsulosin-treated patients undergoing elective cataract surgery.

**Methods:**

Patients using tamsulosin were dilated either with mydriatic-cocktail soaked sponge (group 1) or with conventional eyedrop regimen (group 2). Control patients not taking any α_1_ adrenergic receptor inhibtors were also dilated with mydriatic sponge (group 3).

In all groups oxybuprocain 0.4%, cocain 4%, tropicamide 1%, phenylephrine 10%, diclophenac 0.1% along with chloramphenicol 0.5% were used preoperatively.

Pupil diameter (mm) was measured preoperatively, after nucleus delivery, and before IOL implantation. Adverse effects associated with the use of sponge, minor and major intraoperative complications, the use of iris retractors and operation time were recorded.

Differences in general between groups were analyzed with a one way analysis of variance (ANOVA); differences between groups in proportions were assessed by Fisher’s exact test.

**Results:**

Mean pupil diameter (mm) was preopertively: 7.52 ± 1.21, 7.30 ± 1.55 and 7.99 ± 0.96 (ANOVA: p = 0.079); after nucleus delivery: 6 ± 1.20, 6.29 ± 1.12 and 6.52 ± 0.81 (ANOVA: p = 0.123); before IOL implantation: 5.46 ± 1.06, 5.83 ± 1.09 and 6.17 ± 0.89 (ANOVA: p = 0.0291).

No adverse effect related to sponge use was detected. Frequency of minor complications, and iris hook use was similar in the two tamsulosin treated group. Operation time did not differ significantly in the three groups.

**Conclusion:**

We have found that using a mydriatic cocktail-soaked wick – an alternative way to achieve intraoperative mydriasis for cataract surgery – was as effective and safe as the conventional repeated eyedrops regiment for tamsulosin treated patients.

**Trial registration:**

Current Controlled Trials ISRCTN37834752

## Background

Proper perioperative mydriasis and anaesthesia are prerequisite to successful cataract surgery.

Cataract operation may be performed using different preoperative protocols which should be determined according to the needs and preference of the patient, the anaesthesia professionals and the surgeon [1]. Adequate pupil dilation is even more crucial for patients who are at risk of limited pupillary function.

Intraoperative floppy iris syndrome (IFIS) associated with tamsulosin – a systemic α_1_ adrenergic receptor antagonist (α1-ARA) - was first described by Chang and Campbell in 2005. It is characterised by the triad of (1) flaccid iris stroma that undulates and billows in response to ordinary intraocular fluid currents, (2) tendency for the iris stroma to prolapse towards the phaco and side-port incisions, and (3) a progressive intraoperative pupillary miosis during cataract surgery [2].

IFIS may lead to the constriction of the surgical field which increases vision-threatening complications of cataract surgery such as iris stroma damage, posterior capsular rupture, and loss of vitreous, particularly when surgeons are unaware of patient’s medical history [3-5].

Systemic α-blockers are used to treat the urinary symptoms of benign prostatic hypertrophy (BPH). These agents relax smooth muscle in the prostate and bladder. This effect is utilized to improve symptoms associated with BPH [6,7]. However, this α-blockade can influence iris motility also.

A strong association was found between IFIS and tamsulosin [8] – a selective α_1A_ and α_1D_ adrenergic receptor antagonist, which is a common therapeutic option for BPH. Several studies showed a risk of IFIS as high as 53.3%-93.8% with tamsulosin use [2,9-14].

Various strategies were advocated for patients taking α1-ARA to achieve appropriate mydriasis and to overcome the progressive miosis such as: preoperative topical atropine, intracameral injection of epinephrine, careful wound construction, mechanical pupil dilation, gentle hydrodissection, low-flow fluidic settings, and bimanual irrigation-aspiration [15-17].

Preoperative discontinuation of α_1_ adrenergic receptor antagonist medication has an unclear role in inhibiting the occurernce of IFIS [2,5,12,17-20].

All of the above techniques serve as preventive measures to minimize the risk of IFIS, however they do not eliminate its occurrence.

Previous studies indicated that the use of a wick pre-soaked in standard mydriatic and non-steroidal anti-inflammatory drugs was as effective or superior to the conventional repeated instillation of drops before cataract surgery in a mixed cohort [21-24].

In our study we investigated the efficacy of a mydriatic cocktail-soak sponge as an alternative method to achieve satisfactory pupil dilation in high risk patients taking tamsulosin.

## Methods

### Patients

Male patients taking tamsulosin due to benign prostatic hypertrophy, attending elective cataract surgery at Thy-Mors Hospital (Thisted, Denmark) were enrolled in our study from October 2012 until December 2012 (n = 65). Along with the study group patients we enrolled 31 control males not receiving any α_1_ adrenergic receptor antagonist medication. Medical and ophthalmic history was recorded at the preoperative visit. Previous ocular surgery (1), posterior synechiae (2) and the use of drops other than artificial tears (3) were exclusion criteria (n = 60). The study group was subdivided randomly into two equal sized groups (n = 30), using sealed envelope method. The randomization was carried out by the anaesthetic nurse in the anaesthetic room. Surgeons were masked for the randomisation but not for the use of tamsulosin due to regional safety guidelines. 30 patients were dilated using the mydriatis cocktail-soaked sponge (group 1), and 30 patients were dilated using conventional repeated eyedrops regimen (group 2). We had to cancel two patients from group 2 in the operation room due to fact that they could not lie flat and still. Control patients (group 3; n = 31) were also dilated with mydriatic-cocktail soaked sponge (Additional file 1).

The study adhered to the tenets of the Declaration of Helsinki, and was approved by The North Denmark Region Committee on Health Research Ethics (N-20120049). Written informed consent was obtained from all participants in this study. The patients in the figures gave written consent for publication of their image.

### Preoperative preparation

In all groups oxybuprocain 0.4%, cocain 4%, tropicamide 1% (Minims® Tropicamide, Bausch&Lomb), phenylephrine 10% (Minims® Phenylephrine hydrochloride 10%, Bausch& Lomb), diclophenac 0.1% (Diclofenacnatrium Stulln, Pharma Stulln GmbH, Stulln, Germany) along with chloramphenicol 0.5% eyedrops were used preoperatively.

For the sponge groups (group 1 and group 3) 4 mm × 5 mm pieces of cellulose wicks (BD Visitec™, Eye Fluid Wick, Becton Dickinson, Dublin, Ireland) were prepared and submerged in a mix of the above solutions with 1:1:1:1:1:1 ratio. Sterile mydriatic solution was prepared daily.

**Figure 1:**
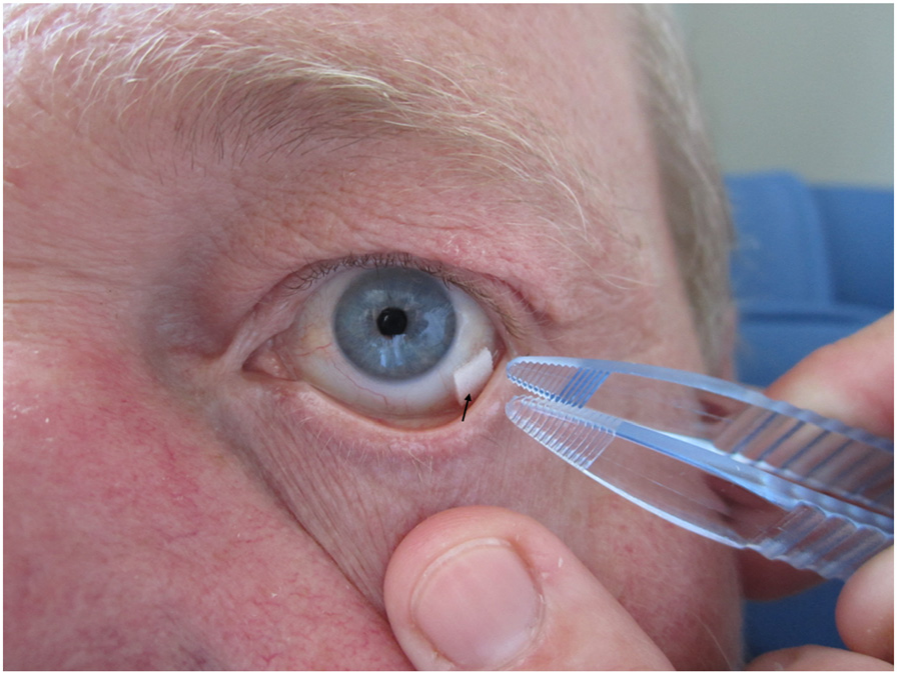
Mydriatic cocktail-soaked sponge inserted in the outer lower fornix (arrow).

**Figure 2:**
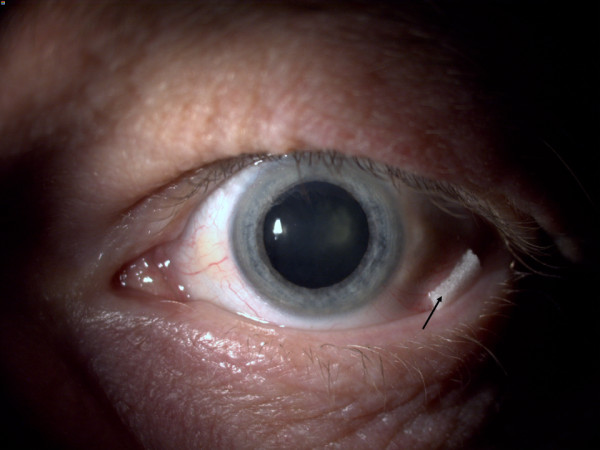
**Assesing pupil dilation after 30 minutes.** Arrow points towards the sponge.

After topical administration of one drop oxybuprocain 0.4% anaesthesia a piece of soaked wick was inserted in the outer lower fornix for 30 minutes (Figures 1 and 2).

Any adverse effect associated with the use of sponge such as corneal abrasion, allergic reaction or wick migrating to the upper fornix was recorded.

In the conventional repeated eyedrops group (group 2) we applied the above drops 3 times with 10 minutes intervals.

Surgeons were not involved in the dilatation process.

### Surgical technique and intraoperative measurements

Patients recieved a drop of oxybuprocain 0.4% before inserting the specula, and before initial wound construction.

An oblique or a temporal 2.75 mm clear cornea, 3-step incision was used along with two 1.5 mm side incisions. After injection of viscoelastic materials (Duovisc, Alcon Fort Worth, TX, USA) for anterior chamber maintenance, a continuous curvilinear capsulorrhexis (CCC) and hydrodissection were performed. The lens was phacoemulsified using either devide-and-conquer or horizontal chop technique and irrigation aspiration was performed bimanually. In each case we implanted a single piece foldable acryllic lens (Biocomfold 92, Morcher, Stuttgart, Germany) in the capsular bag. After complete removal of viscoelastic materials, the clear corneal and side incisions were hydrosealed. At completion of surgery, 1 mg intracameral cefuroxime was administred.

**Figure 3:**
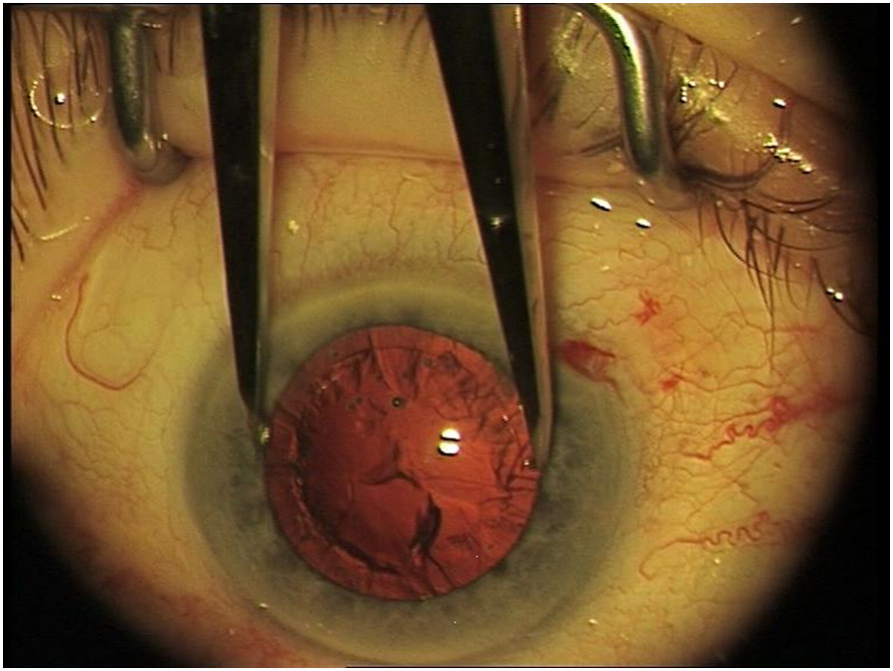
Intraoperative measurement of pupillary diamater with Castoviejo caliper.

Pupil diameter was measured with 0.5 mm increments in the horizontal plain preoperatively, after nucleus delivery, and before IOL implantation using Geuder Castroviejo caliper (Geuder AG, Heidelberg, Germany) (Figure 3). The use of flexible iris retractors (Alcon Laboratories, Inc., Fort Worth, TX, USA) were recorded. In case of iris retractor use, patients were excluded from further pupillary mesaurements, and statistic. Intraoperative miosis was assesed between the preoperative measurement and the measurement after nucleus delivery (miosis 1); between the measurement after nucleus delivery and measurement before IOL implantation (miosis 2); and between the preaoperative measurement and the measurement before IOL implanatation (total intraoperative miosis).

Intraoperative iris billowing, iris prolapse, minor intraoperative complications (iris damage, iris bleeding, retained cortex) and major intraoperative complications (anterior/posterior capsular tear, vitreous loss, nuclear fragment/cortical material loss and zonular dialysis) were recorded.

The presence of IFIS was recorded upon the occurance of any symptom, according to its definition.

Duration of operation was measured from the initial wound construction until removal of the specula.

### Statistical analysis

The size of the study was decided from the following power considerations. With 25 patients in each of two groups, a 0.8 mm difference between groups will be recognized by an unpaired t-test with 80% probability assuming a within group standard deviation of approximately 1 mm.

Differences in general between groups (age, pupil diameter, miosis, and operation time) were analyzed with a one-way analysis of variance (ANOVA), and differences between specific groups (group 1 vs. group 2 and group 1 vs. group 3) were assessed by suitable contrasts assuming normality and variance homogeneity. Mean and 95% confidence intervals are given for each comparison. Normal distribution was assessed by qq plots and variance homogeneity by robust variance test.

Differences between groups in proportions (hypertension, diabetes, clear corneal incision location, IFIS signs, complications and use of iris retractors) were assessed by Fisher’s exact test.

## Results

Table 1 summarizes preoperative data, comparing results from group 1 (patients using tamsulosin, dilated with mydriatic-cocktail soaked sponge) with group 2 (patients using tamsulosin, dilated with conventional repeated eyedrops regimen) and group 1 with group 3 (control patients, dilated with mydriatic-cocktail soaked sponge) respectively.

**Table 1:** Preoperative data

**Patient group and pupil dilation methods**					
	**Group 1. n = 30**	**Group 1 vs. Group 2**	**Group 2. n = 28**	**Group 3. n = 31**	**Group 1 vs. Group 3**
**Mean age ± SD (years)**	78.6 ± 10.35	p = 0.350; 95% CI −6.10-2.18	76.64 ± 6.85	78.48 ± 5.84	p = 0.954; 95% CI −4.15-3.92
**Hypertension**	17/30	p = 1.000	15/28	17/31	p = 1.000
**Diabetes**	10/30	p = 0.243	10/28	6/31	p = 0.518

Group 1. Tamsulosin treated patients and cocktail-soaked sponge dilation.

Group 2. Tamsulosin treated patients and conventional drop dilation.

Group 3. Control patients and cocktail-soaked sponge dilation.

n = number of patients, SD = standard deviation, p = value of significance, 95% CI = 95% confidence interval.

Mean age ± standard deviarion (SD) was 78.60 ± 10.35 years in group 1, 76.64 ± 6.85 years in group 2, and 78.48 ± 5.84 in group 3. One way ANOVA showed no significant age difference between the three groups (p = 0.5777).

No adverse effect related to mydriatic-sponge use was experienced.

Table 2 shows the intraoperative data comparing group 1 with group 2 and group 1 with group 3 respectively.

**Table 2:** Intraoperative data

**Patient group and pupil dilation methods**					
	**Group 1. n = 30**	**Group 1 vs. Group 2**	**Group 2. n = 28**	**Group 3. n = 31**	**Group 1 vs. Group 3**
**Pupillary diameter ± SD (mm)**					
Preoperatively	7.52 ± 1.21	p = 0.492; 95% CI −0.83 - 0.40	7.30 ± 1.55	7.99 ± 0.96	p = 0.124; 95% CI −0.13 - 1.06
After nucleous delivery	6 ± 1.20	p = 0.247; 95% CI −0.23 - 0.87	6.29 ± 1.12	6.52 ± 0.81	**p = 0.042**; 95% CI 0.20 - 1.08
Before IOL implantation	5.46 ± 1.06	p = 0.178; 95% CI −0.17 - 0.90	5.83 ± 1.09	6.17 ± 0.89	**p = 0.008**; 95% CI 0.19 - 1.22
**Miosis 1 ± SD (mm)**	1.52 ± 0.88	p = 0.330; 95% CI −0.64 - 0.22	1.31 ± 0.75	1.55 ± 0.74	p = 0.874; 95% CI −0.38 - 0.44
**Miosis 2 ± SD (mm)**	0.61 ± 0,45	p = 0.349; 95% CI −0.35 - 0.12	0.5 ± 0.47	0.35 ± 0.37	**p = 0.024**; 95% CI −0.39 - 0.03
**Total intraoperative miosis ± SD (mm)**	2.18 ± 0.91	p = 0.073; 95% CI −0.83 - 0.04	1.79 ± 0.67	1.88 ± 0.78	p = 0.156; 95% CI −0.72 - 0.18
**Location of tunnel wound**	t:15 o:15	p = 1.000	t:14 0: 14	t:16 o:15	p = 1.000
**IFIS signs**					
Iris billowing	19/30	p = 0.583	20/28	5/31	**p = 0.000**
Iris prolaps	7/30	p = 1.000	7/28	1/31	**p = 0.026**
**Intraoperative complications**					
Minor	3/30	p = 1.000	2/28	1/31	p = 0.354
Major	1/30	p = 1.000	0/28	0/31	p = 0.492
**Use of iris retractors**	3/30	p = 1.000	2/28	1/31	p = 0.354
**Mean operation time ± SD (minutes)**	15.87 ± 3.21	p = 0.256; 95% CI −0.65-2.42	16.75 ± 2.69	15.26 ± 2.87	p = 0.421; 95% CI −2.10-089

Group 1. Tamsulosin treated patients and cocktail-soaked sponge dilation.

Group 2. Tamsulosin treated patients and conventional drop dilation.

Group 3. Control patients and cocktail-soaked sponge dilation.

n = number of patients, SD = standard deviation, IOL = intraocular lens, t = temporal incision, o = oblique incision, IFIS = intraoperative floppy iris syndrome.

p = value of significance, 95% CI = 95% confidence interval.

Statistically significant differences between groups are highlighted in bold.

One way ANOVA test did not show significant difference in pupillary diameter between groups preoperatively (p = 0.079) and after nucleus delivery (p = 0.123), however we found significant difference before IOL implantation (p = 0.0291).

We found no significant difference between the three groups in miosis 1 (p = 0.4763), miosis 2 (p = 0.0760) and in miosis total (p = 0.1678).

In the two tamsulosin treated groups, the patients dilated with a mydriatic wick had greater preoperative mydriasis in comparison with patients receiving conventional eyedrops regimen, however, this difference was not significant. This slight difference disappeared at later stages of surgery.

There was even distribution of temporal and oblique clear corneal incision in all 3 groups.

In group 1, iris retractors were used before completing nucleus delivery in a single case and two additional cases after nucleus delivery. In group 2, we retracted iris with hooks in two patients after clear corneal incisions. In group 3, we had to facilitate surgery by using iris retractors with a patient before completing nucleus delivery.

IFIS signs were observed in a significantly higher degree in group 1 compared with group 3.

Minor (3/30, 2/28 and 1/31 respectively) and major (1/30, 0/28 and 0/31 respectively) complication rate was similar in all groups.

Mean operation time was shorter in group 3 (15.26 ± 3.21 minutes) than in the two tamsulosin treated groups (15.87 ± 3.21 minutes and 16.61 ± 2.59 minutes) respectively, but the difference was not significant (p = 0.1547).

## Discussion

Cataract surgery is more challenging in patients taking α1-ARA due to poor and unstable perioperative mydriasis [2,6,8,16]. History of tamsulosin use among patients undergoing cataract surgery was estimated around 3% in the United States [25-27]. Although several pre- and intraoperative methods are used to overcome this problem, IFIS remains one of the pitfalls of cataract surgery.

In our study we investigated the efficacy of mydriatic cocktail-soaked sponge pupil dilation in patients using tamsulosin due to benign prostatic hypertrophy. Our results indicated that regardless of the preoperative protocol, patients with tamsulosin treatment show poorer perioperative mydriasis in comparisons with control subjects.

Comparing the two groups having the mydriatic cocktail-soaked sponge pupil dilation preoperatively (group 1 vs. group 3), patients with tamsulosin treatment showed significantly smaller pupil diameters after nucleus delivery and before IOL implantation but not preoperatively.

Patients in group 1 showed significantly higher degree of miosis between our second and third pupil measurement (miosis 2) in comparison with group 3 (p = 0.024). These findings are consistent with the fact that patients with tamsulosin treatment develop progressive intraoperative pupillary miosis during cataract surgery [2,3,10]. In contrast, Casuccio *et al.* found that pupil diameter was significantly smaller both preoperatively and postopoperatively in patients taking tamsulosin compared with their control group, however both exclusion criteria and their mydriatic regimen were different [27].

We had a uniform distribution of oblique and temporal wounds in each group, which limits observational bias between groups. It has been shown that the anterior chamber is deeper superiorly than temporally, and therefore a superior approach may be preferred in some cases to minimize the risk or iris prolapse [28,29]. However there is a general consensus that meticulous wound construction – 2-step or 3-step incision, appropriate lenght of the wound, and the location of entry into the anterior chamber in relation to the iris plane – is essential in preventing intraoperative iris prolapse [29-32].

The incidence of iris prolapse was similar in our two tamsulosin treated group (23% and 25% respectively). This incidence is lower that in the largest prospective study (n = 167) published to date [10], however α1-ARA medication is only one of the many predisposing factors [29]. Iris billowing – a hallmark of IFIS – was found in similar percentage in the two tamsulosin treated group as in two previous studies [11,24].

The frequency of iris retractors use was similar in the two tamsulosin groups (3/30 vs. 2/28), and lower than in the previously pubished results of Blouin *et al.,* and Issa *et al.* 42.6% and 38% respectively [12,33].

Minor complication rate was higher in the two tamsulosin treated groups (10% and 7.1%) than in the control group (3.2%), however this difference was not significants which is probably due to the low sample size. A single patient treated with tamsulosin had posterior capsule rupture without vitreous loss in group 1. Patients having minor or major complications did not have significantly smaller preoperative pupillary diameter. Minor complication rate was similar as in the recently published studies, however lower than in earlier studies; this is probably due to the increasing awareness of IFIS and to our unmasked study fashion [2,3,10,14,27,34,35]. The relatively high incidence of minor complication in the control group might be related to small sample size or to the high prevalance of systemic hypertension, which was proposed as a possible cofounder of IFIS [13].

We use mydriatic cocktail-soaked sponge pupil dilation as a standard method in our cataract surgery protocol. In our practice, the use of mydriatic sponge beside the effective intraoperative mydriasis also gives considerable saving in nursing resources along with medicine expenses, in comparison with conventional repeated eyedrops method. We observed that this method is as effective as the conventional drop regimen in a mixed case cohort (unpubished results), but we have not found any previous publication indicating that this method may be suitable for high risk patients such as patients taking tamsulosin.

Our results show that the sponge was as effective in achiving sufficient periopereative mydriasis in tamsulosin treated patients as the conventional repeated eyedrops method. The use of mydriatic soaked sponge was not associated with any advers effect, and showed similar rate of minor complications as found in the conventional repeated eyedrops group.

Previously, three randomised controlled studies investigated the use of mydriatic drug delivery by a soaked wick placed in the lower fornix [22-24]. In all these studies, similar exclusion criteria were applied as in our study, however, in the first study patients with diabetes and very dark irides, and in the latest study, patients with systemic hypertension and ischemic heart disease were also excluded. These studies did not registered the use of α1-ARA medicines.

Only the study by McCormick indicated a clear timeframe for pupil dilation: the pledget sponge was removed after 20 minutes. In our protocol, we terminated pupil dilation 30 minutes after initiation.

In the first study preoperative pupil diamater was measured 1–4 hours after initiation of pupil dilation with a wick soaked in mydriatic drops (Tropicamide 1%, Phenilephine 2.5% and Diclophenac sodium 0.1%), but no intraoperative measurement was taken. In the second study horisontal pupil diamater was measured in milimeters prior to surgery, and 40 minutes after commencement of mydriatic regime (Tropicamide 1%, Phenylephrine 2.5% and Atropine 1%) without any intraoperative pupil assesment. In the latest study pupillary diameters were measured 15, 30, 45 minutes after placing in the lower fornix a sponge - immersed in the cocktail regiment consisting of 1:1:1:1 ratio of 2.5% phenylephrine, 0.5% moxifloxacin, 1% cyclopetolate and 0.03% flurbiprofen eye drops. Pupil size was also assessed intraoperatively after nucleous delivery and IOL implantation.

Preoperative pupil diameter was higher in the study by Dubois *et al.,* similar to McCormick *et al.* and lower in by Sengupta *et al.* than in our study, however all three studies used different mydriatic cocktail regime and duration of sponge use. We observed similar pupil diameters after nucleous delivery as in the latest study (6.20 mm).

The study by Dubois *et al.* and McCormick *et al.* found that the cocktail-soaked sponge protocol leads to similar results as the conventional drop application. The study by Sengupta *et al.* showed that results were superior with the soaked-wick method.

Our study group of tamsulosin patients had much higher risk of developing progressive intraoperative miosis - a component of IFIS - than a mix cohort. Various studies indicated up to hundred times higher incidence of IFIS in patients with α1-ARA medication. None of the previous three studies investigated the presence of IFIS or intraoperative complications.

Pupil dilation by mydriatic cocktail-soaked sponge showed to be effective in all three studies without any adverse event that might be related to the use of sponge. The sponge primarily used to absorb and drain blood and fluid from the surgical field during ophthalmic procedures, but alternatively it serves as mydriatic and anaesthetic reservoir from which drugs diffuse down along its concentration gradient into the ocular tissues.

Our study has certain limitations. First surgeons were not masked to use of tamsulosin due to institutional quality regulations that require the preoperative checking of patient medication, which may lead to observational bias. Second, the detection of IFIS (iris billowing) relied on surgeon’s subjective assessment, however this limitation is common in most studies, and the incidence of IFIS in our study was similar to previous findings. Third, we had a relatively small sample size which might have an impact on statistical analysis, however the number of tamsulosin treated subjects in our study was similar to most studies published so far.

## Conclusion

In conclusion we have found that using a mydriatic cocktail-soaled wick – an alternative way to achieve intraoperative mydriasis for cataract surgery – was as effective and safe as the conventional repeated eyedrops regiment in tamsulosin using patients. Though this method did not provide clinical difference or benefit compared to the conventional method, it may serve as a safe and timesaving alternative preoperative protocol even for high risk tamsulosin treated patients.

## Competing interests

The authors declare that they have no competing interests.

## Authors’ contributions

JH: study design, operations, acquisition of data, statistical analysis and interpretation of data, manuscript preparation. LV: operations, acquisition of data and interpretation of data. JV: operations, acquisition of data and interpretation of data. FE: operations, acquisition of data and interpretation of data. SLC: statistical analysis and interpretation of data. BH: study design, interpretation of data and manuscript preparation. HV: study design, statistical analysis and interpretation of data and manuscript preparation. All authors read and approved the final manuscript.

## Pre-publication history

The pre-publication history for this paper can be accessed here:

http://www.biomedcentral.com/1471-2415/13/83/prepub

## Supplementary Material

Additional file 1 CONSORT 2010 Flow Diagram. Click here for file
