# Supplementary material for: Comparing the efficacy of mydriatic cocktail-soaked sponge and conventional pupil dilation in patients using tamsulosin – a randomized controlled trial
Source: BMC Ophthalmol. 2013 Dec 20;13:83. doi: 10.1186/1471-2415-13-83 (PMC3878506; doi:10.1186/1471-2415-13-83)
Supplement: Additional file 1 — CONSORT 2010 Flow Diagram. [file 1471-2415-13-83-S1.doc]

**CONSORT 2010 Flow Diagram**

**Allocation**

**Analysis**

**Follow-Up**

**Enrollment**

Assessed for eligibility (n=65)

Excluded (n= 5 )

♦  Not meeting inclusion criteria (n=5)

♦  Declined to participate (n=0)

♦  Other reasons (n=0)

Analysed (n=30)
♦ Excluded from analysis (give reasons) (n=0)

Allocated to intervention: **Mydriatic cocktail-soaked sponge** (n=30)

♦ Received allocated intervention (n=30)

♦ Did not receive allocated intervention (give reasons) (n=0)

Lost to follow-up (give reasons) (n=0)

Discontinued intervention (give reasons) (n=0)

Analysed (n=28)
♦ Excluded from analysis (give reasons) (n=0)

Randomized (n=60)

MS: 1050222216866118
Comparing the efficacy of mydriatic cocktail-soaked sponge and conventional pupil dilation in patients using tamsulosin - a randomized controlled trial

Lost to follow-up (give reasons) (n=0)

Discontinued intervention (give reasons) (Discontinued intervention (give reasons) (n=0)

Allocated to intervention: **Conventional repeated eyedrops regimen** (n=30)

♦ Received allocated intervention (n=28)

♦ Did not receive allocated intervention:

Preoperative restlessness (n=2)
